# High quality VO_2_ thin films synthesized from V_2_O_5_ powder for sensitive near-infrared detection

**DOI:** 10.1038/s41598-021-01025-8

**Published:** 2021-11-05

**Authors:** Xitao Guo, Yonghao Tan, Yupei Hu, Zainab Zafar, Jun Liu, Jijun Zou

**Affiliations:** 1 School of Mechanical and Electronic Engineering, East China University of Technology, Nanchang, 330013 China; 2 Engineering Research Center of Nuclear Technology Application, East China University of Technology, Ministry of Education, Nanchang, 330013 China; 3 National Centre for Physics, Islamabad, 44000 Pakistan

**Keywords:** Materials science, Physics

## Abstract

Vapor transport method has been successfully used to synthesize high quality VO_2_ thin films on SiO_2_/Si substrate using V_2_O_5_ as a precursor in an inert-gas environment. The morphological and structural evolutions of the intermediate phases during the nucleation and growth processes were investigated by SEM and Raman spectroscopy, respectively. The results showed that the conversion of V_2_O_5_ powder to VO_2_ thin films was dominated by a melting-evaporation-nucleation-growth mechanism. Further characterization results demonstrated that the high quality crystals of monoclinic VO_2_ thin films exhibit a sharp resistance change up to 4 orders of magnitude. In addition, the VO_2_ thin films exhibited good near-infrared response, high stability, and reproducibility under ambient conditions, which should be promising for sensitive near-infrared detection. Our work not only provided a simple and direct approach to synthesize high quality VO_2_ thin films with distinct phase transition properties but also demonstrated the possible infrared sensing application in the future.

## Introduction

Being a strong electron-correlated material, vanadium dioxide (VO_2_) undergoes a reversible metal-to-insulator transition (MIT) near 340 K, companying by structural transformation from a low temperature monoclinic (M) form to a high temperature rutile (R) phase^1^. The driving force for the phase transition has been a long-standing dispute, whether the V–V pairing and the unit-cell doubling causes the additional splitting of the 3d_*II*_ band (i.e. Peierls transition)^2,3^, or the opening of a correlation gap originated from carrier localization (i.e. Mott transition)^4,5^. The underlying physical picture of the MIT process has been studied by both theoretical and in situ experimental studies^6–8^. Moreover, the change in crystal structure is simultaneously accompanied by colossal changes in the electrical^8–10^, optical^11–14^, magnetic^15^, and thermal properties^16,17^. These properties make VO_2_ promising in applications in smart windows^14,18,19^, optical modulators^20^, gas sensors^21^, memory devices^22^, photodetectors^23^ and so forth^24,25^. Furthermore, in order to optimize their practical applications, the synthesis of high quality VO_2_ (M) is highly desirable. During the past decades, great progress has been made in controlling the synthesis of VO_2_ structures with different morphologies and related phase transition properties. Different techniques such as chemical vapor deposition^10,21,23,26,27^, physical vapor deposition^15,28^, atomic layer deposition^29^, and hydrothermal method^9,11,30–32^ has been used to synthesize VO_2_ micro-and nanostructures including micro- and nanowires/beams/nets or nanoparticles. On the other hand, magnetron sputtering^8,12–14^, pulsed laser deposition^22,33^ and molecular beam epitaxy^34^, are usually used to synthesize epitaxial films of VO_2_ (constructed by the nanoparticles). Various factors that affect the MIT behavior of the resultant VO_2_ have also been extensively investigated, including size^35^, morphology^2,12,13^, oxygen defect^22,36,37^, hydrogen^15^, and chemical doping^17,30,38^, etc.

However, the solution-based synthesis methods requires a series of complicated reactions and the resultant products are mainly based on B phase VO_2_ nanostructures^39^. On the other hand, the vapor-based depositions need seed promoters such as vanadia precursor (serve as pre-existing nuclei) or a reactive oxygen gas is required to promote the sublimation of vanadia precursor^28,40–42^. In this regard, a simple and direct synthesis of VO_2_ (M) thin films is highly desired. Additionally, despite the increasing research interest in VO_2_, somehow the completer picture of growth mechanism is still not clear. Studies reveal that the ex-situ spectroscopic investigations of initial phases of the growth of VO_2_ where the liquid droplets of V_2_O_5_ nucleate and then these droplets act as nucleation sites for the growth of quasi-1D VO_2_ structures^28,43,44^. Direct in situ observation of VO_2_ nanowires growth using optical microscopy showed that V_2_O_5_ droplets transform into the intermediate V_6_O_13_ nanowires initially and then reduces to form VO_2_ nanowires^45^. However, little attention has been paid to the nucleation and growth processes of VO_2_ thin films. In addition, as a narrow-band gap semiconductor (~ 0.65 eV), VO_2_ should be a suitable material for infrared detectors. In order to realize its practical application, infrared photodetection of VO_2_ thin films need to explore in detail.

In this paper, we present a simple and direct method to synthesize high quality VO_2_ thin films by using V_2_O_5_ powder as a precursor via vapor transport in an inert-gas environment. The conversion processes of V_2_O_5_ powder to VO_2_ thin films are identified as the melting and evaporation of V_2_O_5_ precursor (forming droplets on the SiO_2_ surface), the nucleation of VO_2_ crystals in V_2_O_5_ droplets, the growth of VO_2_ thin films. The high and pure crystal quality of the as-synthesized VO_2_ thin films is demonstrated by Raman spectroscopy, X-ray diffraction (XRD), and X-ray photoelectron spectroscopy (XPS) exhibiting an obvious resistance change up to 4 orders of magnitude and a very small hysteresis across the MIT. High quality VO_2_ thin film has many potential applications, as exemplified by its eminent suitability for near-infrared detector with fast response speed and high stability in atmospheric condition at room temperature.

## Results and discussion

**Figure 1:**
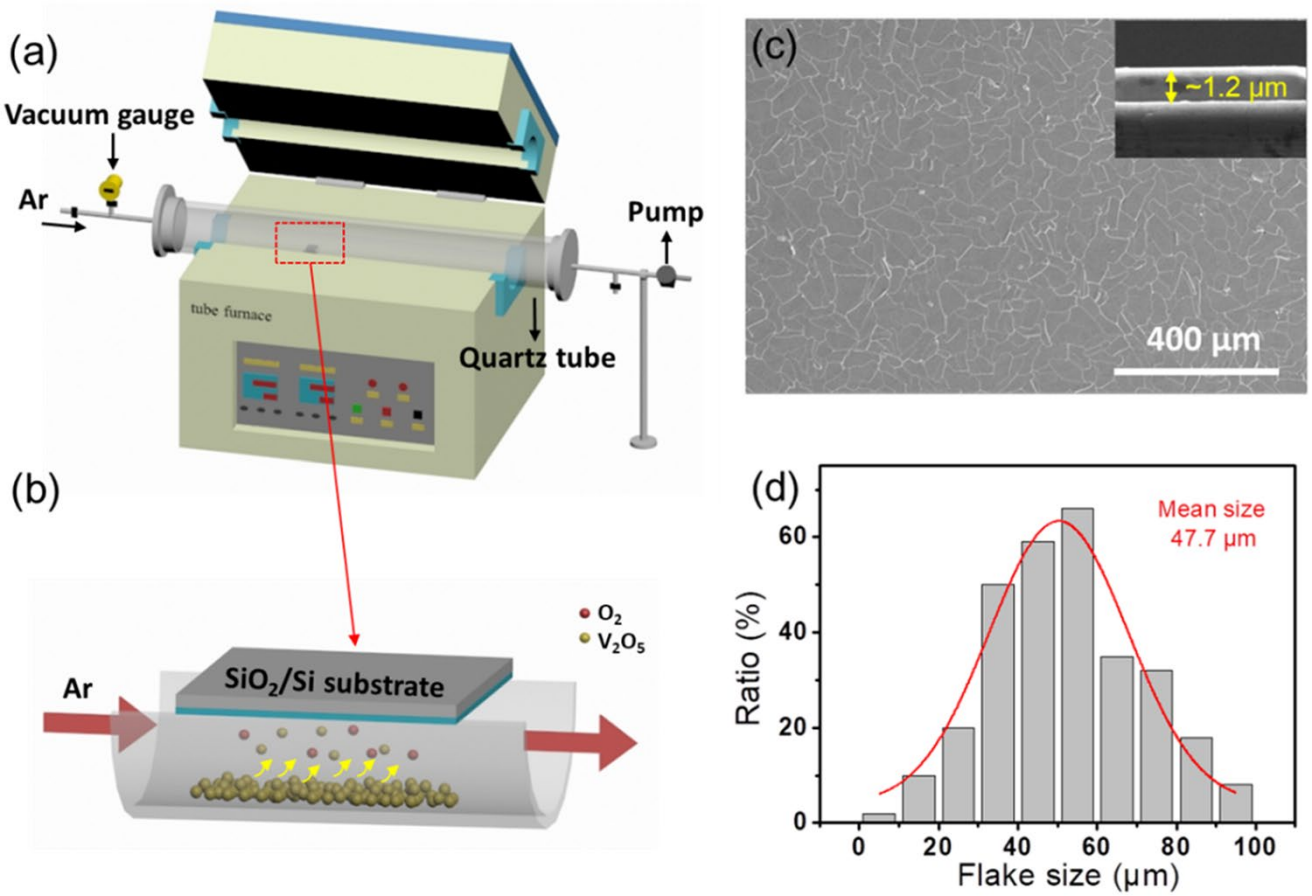
**(a)** Schematic diagram of the tube furnace reactor. **(b)** An enlarged diagram of the quartz boat loaded with V_2_O_5_ powder and growth substrate. **(c)** Surface- and cross-sectional (the inset) SEM images of a complete VO_2_ thin film. **(d)** A histogram of the size distribution of microplates in the VO_2_ thin film.

Figure 1a shows a schematic setup of vapor transport system used in the VO_2_ thin films synthesis. The substrate was upside down over the quartz boat, as shown in Fig. 1b. Figure 1c shows a typical SEM image of a complete VO_2_ thin film, which reveals that the as-synthesized film consists of smooth with irregular shaped grains (on a large scale) called microplates, the connectivity between these irregularly-shaped microplates is quite good. The inset of Fig. 1c shows the cross-sectional morphology of the thin film with a thickness of about 1.2 μm. As shown in Fig. 1d, the mean lateral size of microplates is about 47.7 μm as clearly seen from the size distribution of microplates in a statistical histogram. Histogram has been plotted by measuring the largest dimension of about two hundred microplates in a given image. The dimensions of VO_2_ thin films proved to be sensitively dependent on the synthetic parameters (e.g. precursor flux, not shown here). Additionally, it should be mentioned that because of multivalent of V atom, its oxides can exist in a wide range of stable and metastable stoichiometries that are mutually transformable at specific synthetic conditions as previously reported^45^. Accordingly, the reductive growth of the VO_2_ micro- and nanostructures from the vanadia precursor is considered to be a multistep process, which is triggered by the interplay between the kinetics of vanadia reduction and the thermodynamic stability of different phases^45^.

**Figure 2:**
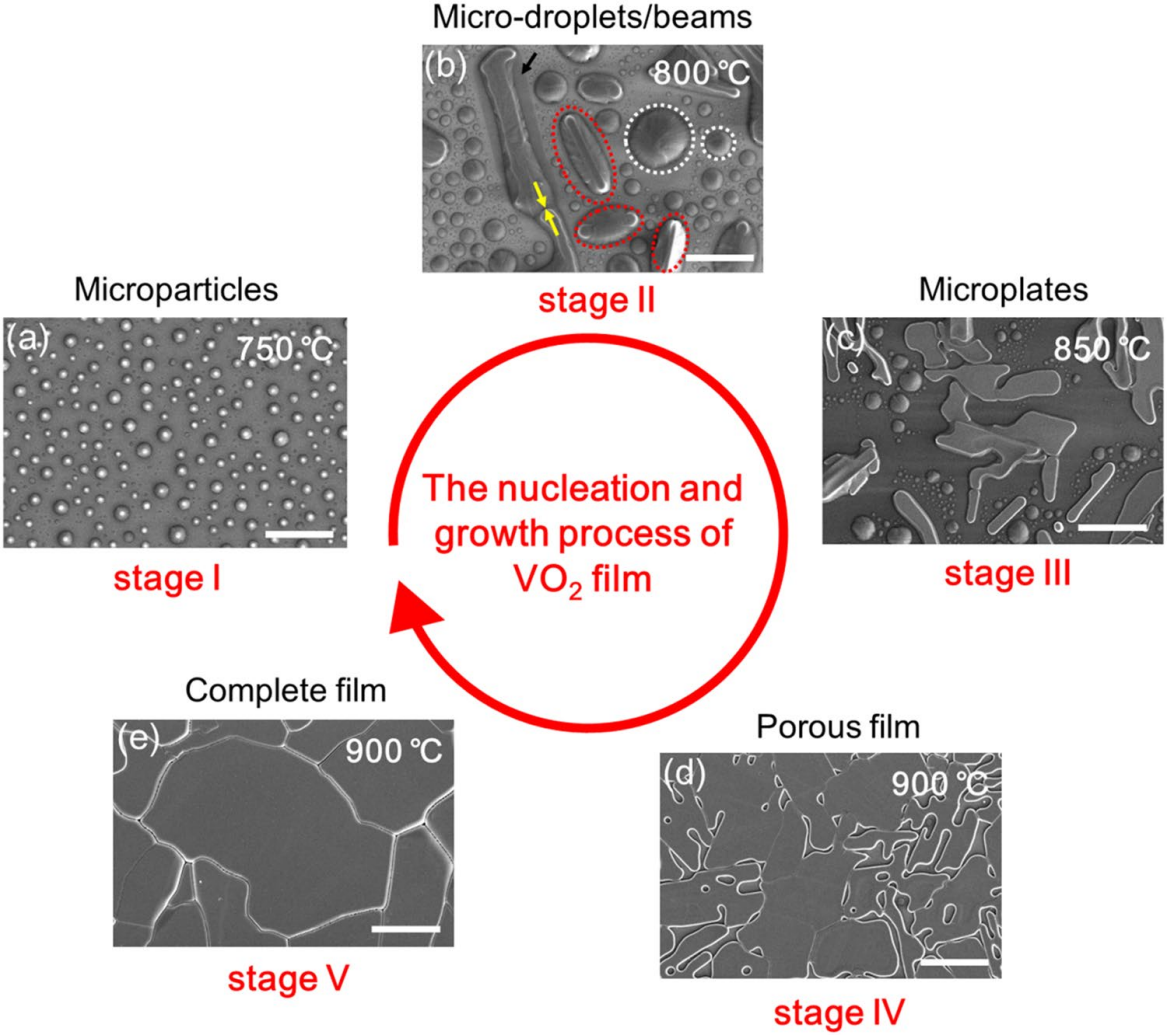
**(a–e)** Representative SEM images of the growth products from the initial stage I to the final stage V, the scale bars in panels correspond to 20 μm.

Considering this, first we perform ex-situ investigations on the surface morphological evolution of the VO_2_ thin film during its nucleation and growth process using SEM. As noted above, the samples at different growth stages are achieved at different temperatures during the thermal ramping in argon flow. The corresponding surface morphologies during the conversion process are shown in Fig. 2a–e. It is expected that initially the fine powder of V_2_O_5_ starts to melt down near its melting temperature (~ 690 °C), and subsequently evaporates from the quartz boat when the furnace is ramped up to 750 °C, forming small droplets (cooled to be microparticles) on the SiO_2_ surface (stage I), as shown in Fig. 2a. Upon further precursor deposition and temperature rise (800 °C, stage II), the small microparticles aggregate melt consistently into large droplets (circled in dotted line), which may occur because of the surface diffusion at higher temperatures results in greater aggregation by Ostwald ripening^45^. Particularly, it is important to note that some parts of the droplets already contain crystals of the guest phase (microbeams, circled in red dotted line), which further undergo a coalescence process that enlarges the width and the length of the microbeams (shown by the yellow arrow). There are obvious traces of grooves left aside around the microbeams due to the dissolution of SiO_2_ during the growth process. When heated up to 850 °C (stage III), one can observe that the microbeams are converted to quasi-2D microplates morphology and the density of these droplets reduces in the vicinity of the microplates, as shown in Fig. 2c. This can be attributed to the fact that high growth temperatures favor the fusing of microbeams along with feeding from the neighboring droplets and enhance their lateral growth. At even higher temperature (900 °C), the growth of single-crystal quasi-2D plates continues until they meet each other, namely, these plates stretch and merge to form a porous film (stage IV, Fig. 2d). After 5 h annealing at 900 °C, the porous film eventually crystallizes and transforms into a complete polycrystalline film due to mass transport by surface diffusion (stage V, Fig. 2e).

**Figure 3:**
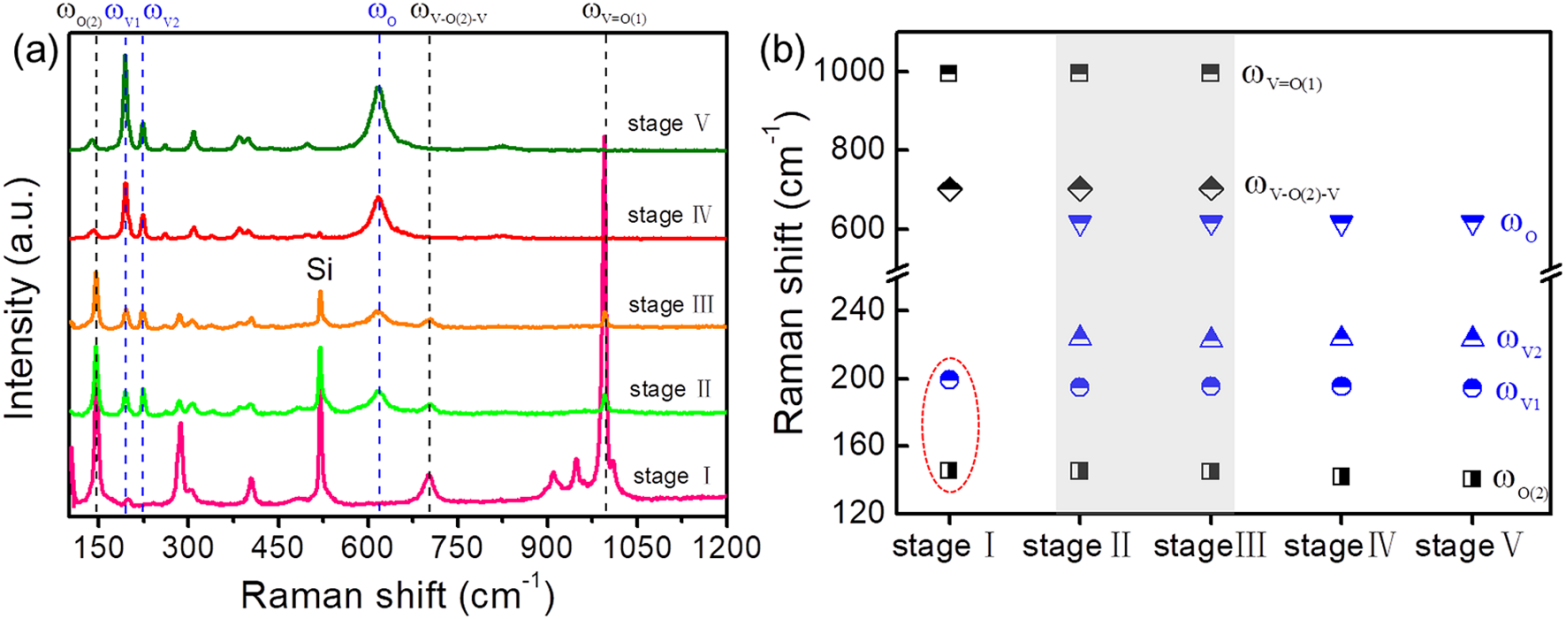
**(a)** Room-temperature Raman spectra of the growth products at different growth stages. Vertical black and blue dotted lines are the three dominant references Raman peaks of V_2_O_5_ and VO_2_ (M) structures, respectively. **(b)** Position evolution of these six Raman peaks during the entire growth process.

It is demonstrated that Raman technique is a powerful method to identify phase structure due to its high sensibility of crystalline lattice variation of vanadium oxides^46^. To complement the morphological studies, the phase structural evolution of the corresponding sample is identified by recording room temperature micro-Raman spectra. Group theory predicts that there are 21 allowed Raman-active modes (14A_g_ + 7B_g_) for the α‐V_2_O_5_ structure and 18 Raman-active vibrations (9A_g_ + 9B_g_) for the VO_2_ (M1) structure, but there is a difference in numbers of experimentally observed Raman-active modes of vanadium oxides due to the different synthetic strategies and polymorphs^46^. In our work, experimentally observed positions of the Raman‐active modes are summarized for all stages of growth in Table 1 (extracted from Fig. 3a). Despite this, the Raman spectrum is typical of α‐V_2_O_5_, with its main features at 147, 285, 305, 405, 702, and 995 cm^−1^, whereas the monoclinic VO_2_ phase has a set of strong well-resolved bands, located at 194, 224, 262, 310, 387, and 612 cm^−1^^46^. Among these, the mode at 147 cm^−1^ (ω_o(2)_) is associated with the in‐phase oscillation of vanadium (shear-like distortions), the band at 702 cm^−1^ (ω_v-o(2)-v)_) is assigned to the asymmetric stretching of V–O_(2)_–V bridges, and the highest intensity feature at 995 cm^−1^ (ω_v=o(1)_) corresponds to the stretching vibrations of V=O_(1)_ vanadyl bonds in V_2_O_5_^28,36,44,46^. Besides, the low-energy peaks at 194 and 224 cm^−1^ (ω_v1_ and ω_v2_) correspond to the motion of vanadium atoms along the c‐axis of the VO_2_ crystal (stretching motion of V–V dimers), while the high-energy one at 612 cm^−1^ (ω_o_) is associated with the V–O vibrations^32,36,46^. We subsequently compare these data with our own experimental Raman spectra to examine the samples' phase structure of each stage, as shown in Fig. 3a. The Raman response of small droplets in stage I demonstrates the typical features of V_2_O_5_ phase, with its main bands at 145, 285, 404, 702 and 994 cm^−1^. The spectrum of microbeams in stage II is similar to that of microplates in stage III, both modes have six characteristic peaks at 145, 195, 223, 617, 703, and 995 cm^−1^. The 195, 223, and 617 cm^−1^ peaks are characteristic Raman modes for monoclinic VO_2_, and the rest three features are attributed to V_2_O_5_ phase, which indicates the formation of mixed VO_2_ and V_2_O_5_ phases at the intermediate stages of growth. A more direct observation of the evolution of these six peaks during the entire growth process is presented in Fig. 3b. In stages IV and V, the film structures are determined to be mostly VO_2_ by their main Raman bands found at 195, 223, 309, and 617 cm^−1^. Additionally, it is important to notice that the relative intensities of the lines at 145, 702, and 995 cm^−1^ are substantially decreased or even disappeared from the initial stage to the final stage, whereas the relative intensities of the lines at 195, 223, and 617 cm^−1^ are noticeably increased. This is a result of the significant increase in relative concentration of VO_2_ crystals in the resultant film.

**Table 1:** Summary of the experimentally observed positions of the Raman‐active modes of the growth products at different growth stages.

Mode	Microparticles	Micro-droplets/beams	Microplates	Porous film	Complete film
B_g_	145.3	145.1	144.8	141.8	140.3
A_g_	199.3	194.8	195.3	195.2	194.4
A_g_		224	222.9	223.6	223.5
B_g_		261	260.3	260.4	260.2
B_g_	285.3	283.8	283.9		
A_g_	305.5	307.2	306.4	309.2	309.4
B_g_		339.9	339.1	339.6	338.4
A_g_		385.6	384.1	384.7	384.5
A_g_	404.5	404.9	404.4	400.6	400.3
A_g_				497.3	498.8
A_g_		617.4	617.5	617.2	617.8
A_g_	701.8	703.3	703.6		
B_g_				825.3	824.6
B_g_	909.5				
A_g_	947.5				
A_g_	994.3	995.8	995.4		
A_g_	1009.9

Combining the above results on morphological and structural changes, the conversion processes of VO_2_ thin films from V_2_O_5_ powder can be summarized as follows. Initially V_2_O_5_ powder starts to melt down in the vicinity of its melting temperature, and subsequently evaporates from the quartz boat when the temperature rises, forming small V_2_O_5_ droplets on the oxide substrate. Later, VO_2_ nucleates inside the V_2_O_5_ droplets and the nuclei grow into microbeams, and theses beams further stretch to form microplates at higher temperature by feeding from the neighboring V_2_O_5_ droplets. As the temperature further rises, the irregular quasi-2D microplates join together to form a porous VO_2_ thin film, and finally crystallize into a complete VO_2_ thin film after a long duration. Also, the thermal decomposition of V_2_O_5_ powder of the as above the discussed phase transformations can be described as

$${2\mathrm{V}}_{2}{\mathrm{O}}_{5}\left(\mathrm{s}\right)\to {2\mathrm{V}}_{2}{\mathrm{O}}_{5}\left(\mathrm{l}\right)\to {2\mathrm{V}}_{2}{\mathrm{O}}_{5}\uparrow \left(\mathrm{g}\right)\to {2\mathrm{V}}_{2}{\mathrm{O}}_{5}\left(\mathrm{l}\right)\to 4{\mathrm{VO}}_{2}(\mathrm{s})+{\mathrm{O}}_{2}\uparrow (\mathrm{g})$$

**Figure 4:**
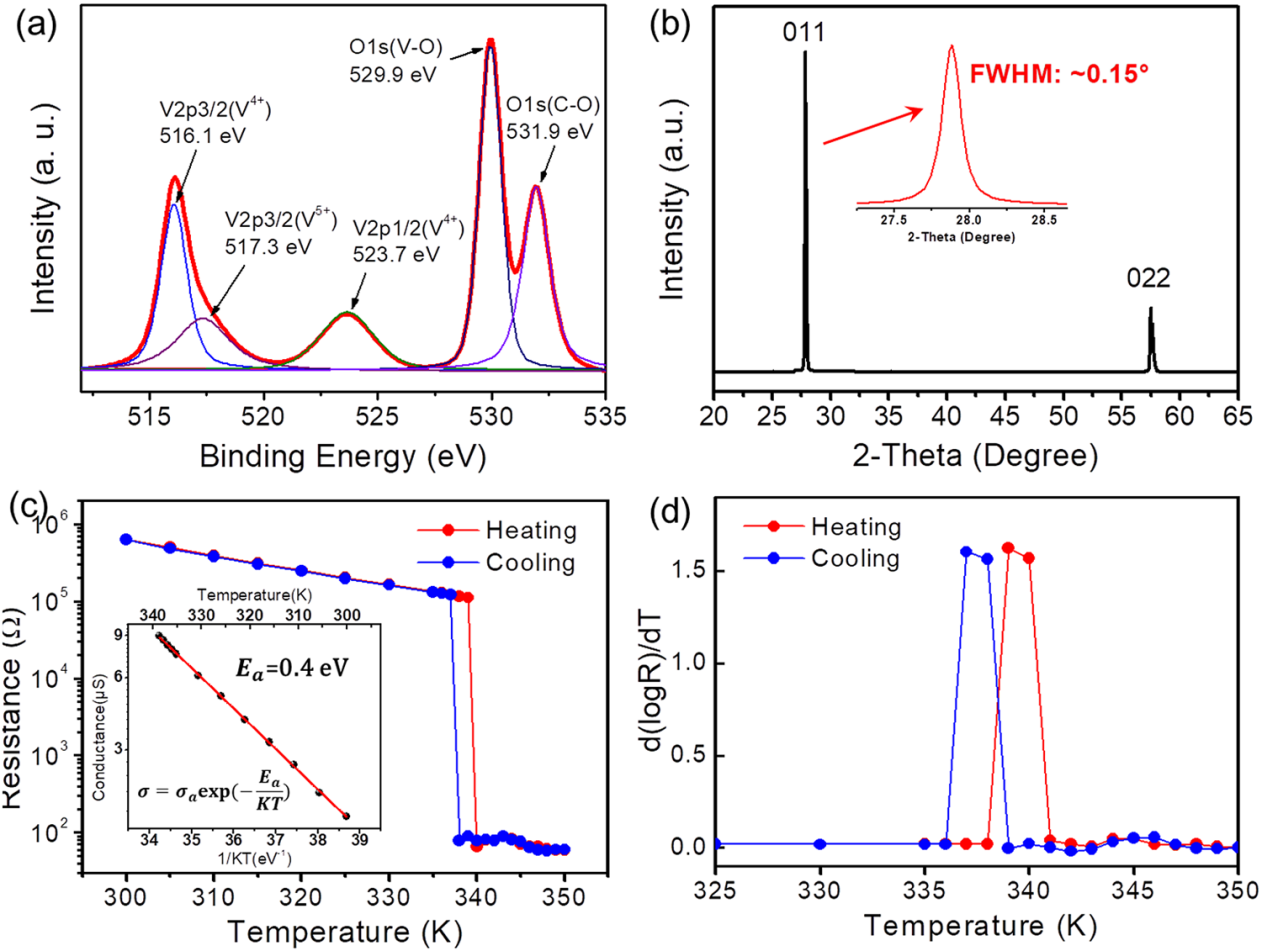
**(a)** High-resolution XPS profile of V 2p and O 1 s and **(b)** XRD pattern collected from the final VO_2_ film structure. **(c)** Temperature-dependent resistance measurements of the final VO_2_ film structure, and (the inset) conductance as a function of inverse temperature showing an activation energy of 0.4 eV. **(d)** The derivation curve during the heating and cooling ramps.

In addition to Raman analysis, chemical composition data are desired to ensure that the final phase consisted mostly of VO_2_. Figure 4a shows high resolution XPS spectrum of V 2p and O 1 s collected from the final film structures. The V 2p spectrum shows the main peaks of 2p_3/2_ centered at 516.1 eV and 2p_1/2_ centered at 523.7 eV, which corresponds to the V^4+^ oxidation state^11,17,47^. The small shoulder peak at 517.3 eV is attributable to V^5+^ ions due to surface oxidation of the VO_2_ thin films or the presence of minor component of V_2_O_5_^11,17,47^ The O 1 s spectrum can be deconvoluted into two peaks at 529.9 and 531.9 eV, which correspond to VO_2_ and CO_2_, H_2_O, respectively. In our case, the XPS results are consistent with the Raman results. The analysis of the crystal structure is further studied by XRD, as shown in Fig. 4b. XRD patterns of the film confirms the presence of highly crystalline VO_2_ structure, exhibiting two indexed peaks (JCPDS 82-0661), (011) and (022) respectively, located at ~ 27.9° and ~ 57.5° with narrow full width half maximum of ~ 0.15° and ~ 0.3°, consistent with the monoclinic VO_2_ structure^17,26^. The strong diffraction peak at ~ 27.9° (011) of monoclinic VO_2_ suggests that the film has a preferential orientation (011). It is reported that highly (011) orientated monoclinic VO_2_ thin film in the insulator state is considered to result in an excellent phase transition performance^26^. To verify this, we measure the temperature dependent electrical resistance from a VO_2_ thin film device. As shown in Fig. 4c, with increasing temperatures, the two-terminal resistance gradually decreases, exhibiting the classical activated semiconductor behavior and switches to metallic behavior for temperature above 339 K. Upon cooling, the film displays a reverse jump to the insulating phase at temperature of 337 K, thus a very narrow hysteresis is exhibited. There is a dramatic change in resistance ($\Delta R={R}_{300 K}/{R}_{350 K}$) by over four orders of magnitude over the MIT, which shows comparable or better phase transition properties than that of VO_2_ thin films synthesized by molecular beam epitaxy^34^ and magnetron sputtering^8,12,35^. The inset of Fig. 4c shows the temperature-dependent conductance of the device followed the Arrhenius behavior well with a thermal activation energy (${E}_{a}$) of 0.4 eV^48^. Figure 4d is the first derivative of the temperature curves ($\left|d\left[\mathit{log}\left(R\right)\right]/dT\right|$) in Fig. 4c for a clear view of the phase transition temperature of the thin film.

**Figure 5:**
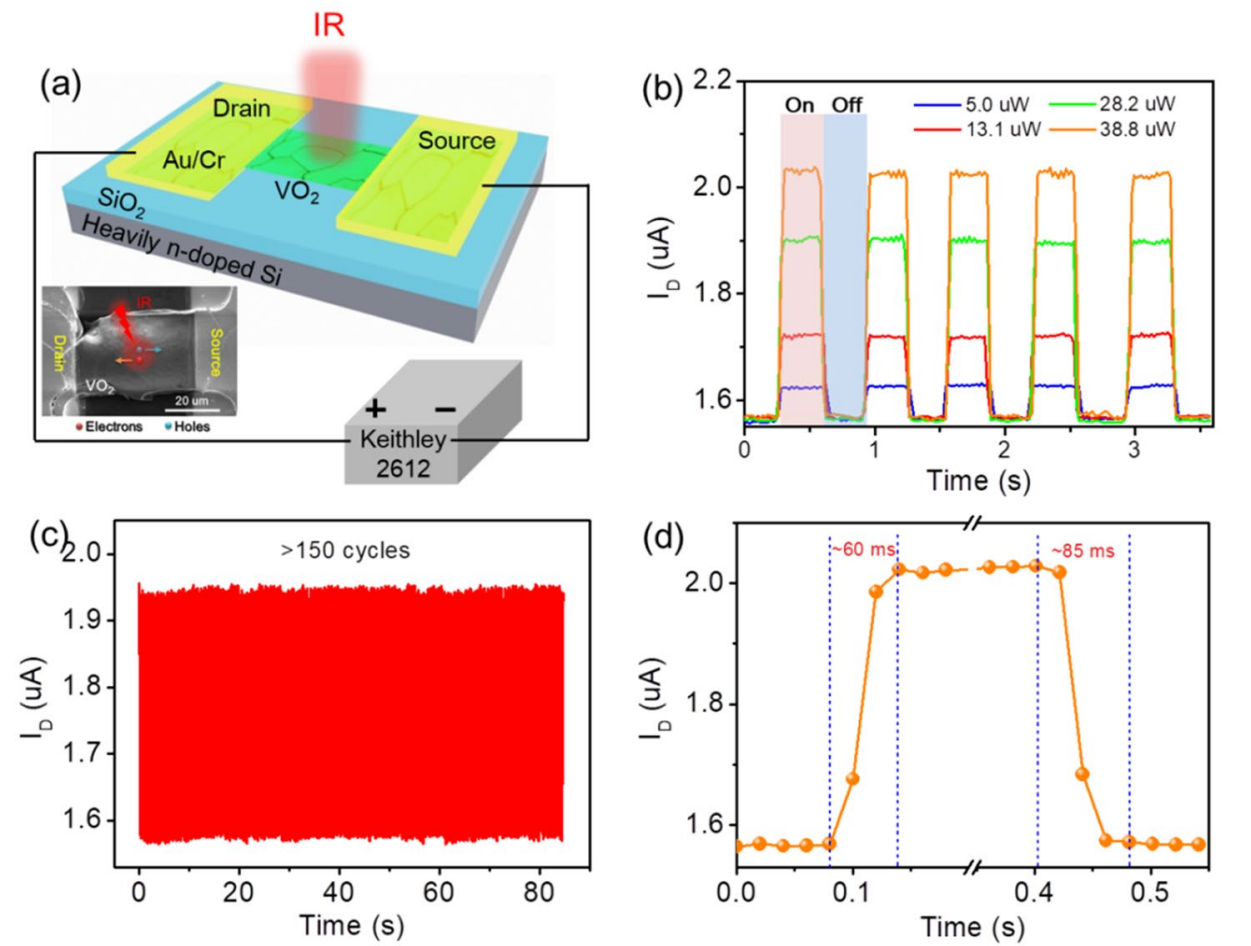
**(a)** Schematic diagram and SEM image of the VO_2_ film IR (850 nm) photodetector. **(b)** Photoswitching characteristics of the IR photodetector under varying light power. **(c)** Repeated photoswitching characteristics of the IR photodetector with more than 150 cycles. **(d)** A single on/off cycle for estimating the rise and fall times.

In case of VO_2_ thin films, most studies in the literatures have been mainly focused on their synthesis and related applications in smart coatings^18^, optical switching devices^49^, laser protective materials^50^, thermal stealth materials^51^ and sensors^21^. However, little attention has been paid to demonstrate the viability of VO_2_ thin films in efficient IR photodetectors. In this work, we investigate the photoelectron characteristics of the as-synthesized VO_2_ thin film under the illumination of an IR light (850 nm). Figure 5a shows the schematic diagram and a representative SEM image of our device. Figure 5b shows that the room-temperature IR response of the channel current under chopping light conditions at a bias voltage of 5.0 V, where a high-current state and a low-current state are observed when light is switched on and off, respectively. Under illumination, the photocurrent (${{I}_{ph}=I}_{light}-{I}_{dark}$) increases rapidly, and then decreases to its initial level in the dark. This is understandable that VO_2_ structure exhibits semiconductor state at room temperature, which can absorb the IR photons and excite photoelectrons from valence band to conduction band, producing an efficient IR response. It should be reminded that the photoresponse can be influenced by the surface morphology of VO_2_ thin films, such as grain boundary density and surface roughness because of their influences on the separation and collection of photogenerated carriers. It can be seen in Fig. 5c that the currents of “on” and “off” states remain almost unchanged for more than 150 cycles, demonstrating that our device exhibits high reversibility and stable characteristics. For the IR photodetectors, the stability and response time are two of the most critical parameters. Response times (rise and fall times) are defined as times required for the current to increase from 10 to 90% or decrease from 90 to 10% of the maximum photocurrent upon on/off cycling^30^. Accordingly, the rise and fall times are respectively measured as 60 and 85 ms from a single on/off cycle (shown in Fig. 5d), showing that the fabricated photodetector was superior to the previously reported IR photodetectors based on VO_2_ nanostructures (e.g. nano-rods/clusters^31,32,47^, and microwire^27^), which exhibit rise and fall processes on a time scale of seconds. The fast response is attributed to the large single-crystal size and good connectivity of the microplates in VO_2_ thin films, demonstrating that our VO_2_ thin films are suitable as IR photodetectors.

**Figure 6:**
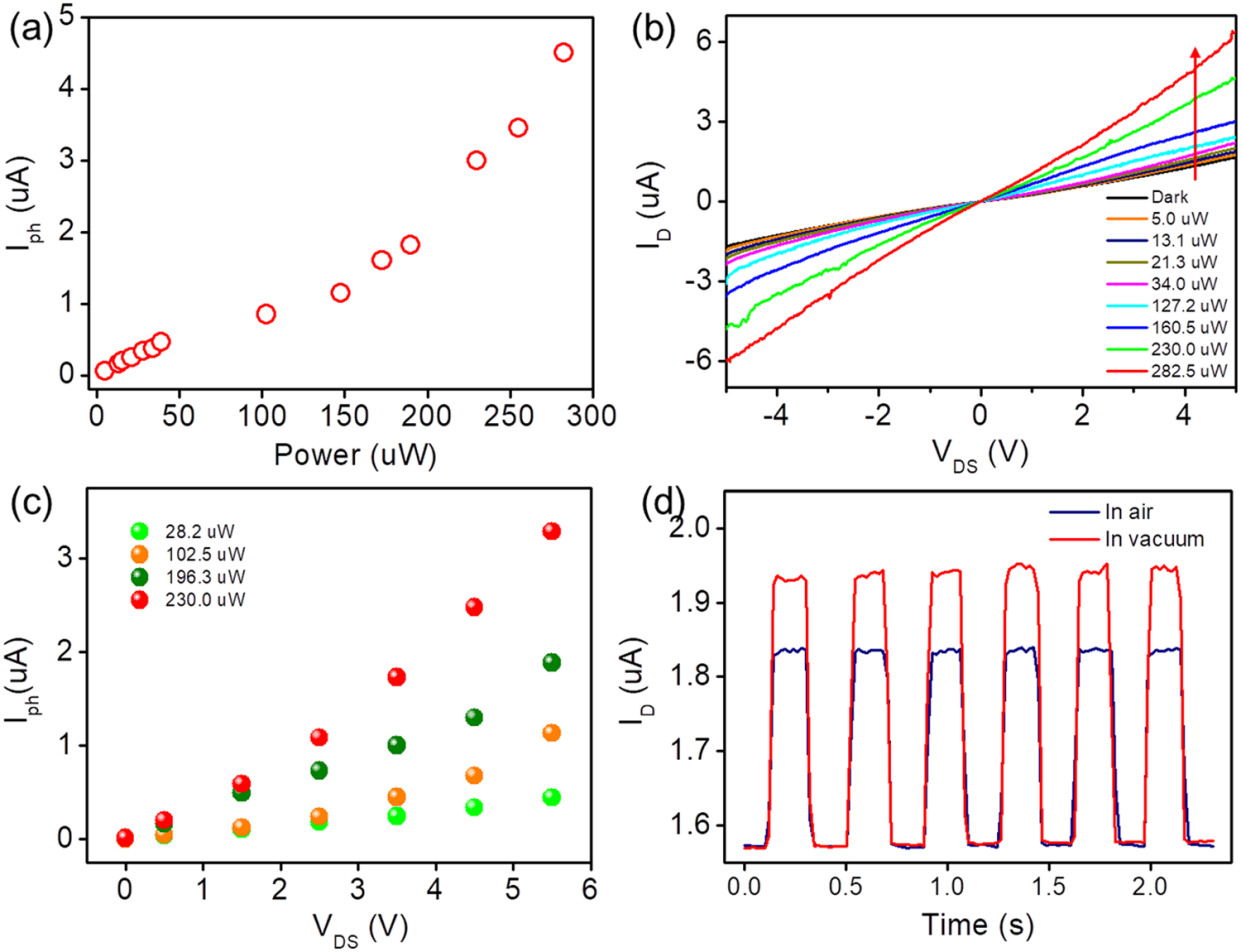
**(a)** Photocurrent versus light power plot at bias of 5.0 V. **(b)** I–V curves of the device at different IR radiation intensities. **(c)** Photocurrent versus bias voltage under varying light power. **(d)** Time-dependent photoresponse of the device in vacuum and air.

Furthermore, the dependence of the photocurrent as a function of light power at a bias of 5.0 V is shown in Fig. 6a. It can be seen that the photocurrent increases gradually with an increase in the light power, and simultaneously deviates linearly with the light power, which is due to the high trap state density (between the Fermi level and conduction band edge of VO_2_)^31,32^. According to the formula ${R}_{\lambda }={I}_{ph}/P$ ^52,53^, where ${R}_{\lambda }$ is responsivity, ${I}_{ph}$ is the photocurrent and $P$ is the power intensity of incident light, a highest responsivity of our device is calculated to be around 16 mA/W. Although there is still room for improvement in responsivity compared to previously reported semiconductor IR photodetectors^54,55^, the values of photocurrent are comparable or higher than that of other previously reported VO_2_ nanostructures-based photodetectors^27,31,47^. Figure 6b shows the current–voltage (I–V) characteristics of the photodetector under dark and light (850 nm IR radiation of different intensities) conditions, revealing that the observed I–V curves exhibit almost linear, thus indicating that the Au electrodes made Ohmic contact with the film and that the junction resistance was relatively smaller than the total resistance of the device. Figure 6c shows the dependence of the photocurrent with varying individual bias voltage under different light powers. The photocurrent significantly increases under illumination, particularly at high bias voltage. The above results imply that the photoresponse characteristics of our device can be effectively tuned by light intensities and bias voltages. Figure 6d shows dynamic photocurrent measured in vacuum and air at light power of 22 μW and bias voltage 5.0 V. The photocurrent is enhanced obviously in vacuum, suggesting that conductance could be increased by decreasing ambient pressure, which is related to a surface oxygen adsorption–desorption mechanism as previously reported in VO_2_ nanowires-based devices^27,30–32^. Overall, the IR response of the VO_2_ thin film device can be understood from the point view of a traditional photon-induced carries transport process (see the inset of Fig. 5a)^47^. Upon 850 nm light illumination, the photons will be absorbed by VO_2_ because of its ~ 0.65 eV bandgap, and electrons will be directly excited from valence band into conduction band, leaving the holes in valence band. The electrons and holes will be further separated by applying bias voltage on the terminal of electrode, resulting in the enhanced channel current and causing efficient IR response.

## Conclusions

In summary, we reported a simple and direct approach to synthesize high quality VO_2_ thin films by the reduction of V_2_O_5_ powder in argon gas flow, and the growth mechanism of the resultant films was systematically examined by interrupting the growth at different temperatures. SEM and Raman spectroscopy were employed to distinguish the morphological and structural changes of the thin films at different growth stages, respectively. Stated simply, the conversion processes of V_2_O_5_ powder to VO_2_ thin films were identified as the melting and evaporation of V_2_O_5_ precursor (forming droplets on the SiO_2_ surface), the nucleation of VO_2_ crystals in V_2_O_5_ droplets, the growth of VO_2_ thin films. XPS and XRD analyses collectively demonstrated that the film structures have high and pure crystal quality of monoclinic VO_2_ phase. Furthermore, a two terminal device structure was fabricated to study the MIT behavior and IR response. The results showed that the VO_2_ film exhibited a dramatic change in resistance by 4 orders of magnitude and a very small hysteresis across the MIT. Compare to previously reported IR photodetectors based on VO_2_ nanostructures (grown by more complicated techniques), the fabricated photodetector exhibited low cost and high-performing IR response with fast response speed (rise and fall times were 60 and 85 ms), and high stability (more than 150 cycles) in atmospheric condition at room temperature.

## Methods

### Synthesis of VO_2_ thin films

Commercial V_2_O_5_ powder (99.99%, Sigma-Aldrich) was used as the vanadium source for the growth of VO_2_ films. Prior to VO_2_ films growth, the 500 nm SiO_2_/Si substrate was ultrasonically rinsed in acetone, ethanol, and deionized water to remove contaminants. The fine powder was loaded on a quartz boat positioned at the center of temperature regions of the tube furnace, and the substrate was upside down over the quartz boat. The amount of precursor was 0.1 g, flow rate of argon was 5 sccm, the pressure was 10 Pa, and the distance between the SiO_2_ surface and the bottom of boat was about 1 cm. The samples at different growth states were obtained when ramped up to 750 °C (stage I), 800 °C (stage II), 850 °C (stage III) and 900 °C (stage IV) at a rate of ~ 15 °C/min, and then cooled to room temperature at a rate of ~ 5 °C/min, respectively. For the growth of a complete VO_2_ polycrystalline thin film (stage V), the temperature was kept constant at 900 °C for 5 h.

### Morphology and structure characterization of VO_2_ thin films

The morphologies and structures of samples at different growth stages were obtained using a field-emission scanning electron microscope (Nova NanoSEM 450). Raman spectra were conducted using a Renishaw micro-Raman system 2000 spectrometers with a wavelength of 532 nm. To avoid unintentional heating during Raman analysis, the incident laser power is limited to 0.2 mW. XRD spectrum was collected using a Bruker D8 Advance scanning X-ray diffractometer equipped with a monochromatic source of Cu Kα radiation at 1.6 kW (40 kV, 40 mA). X-ray photoelectron spectroscopy (XPS) was performed with a Thermo Scientific Escalab Xisystem using Al Kα radiation, and the XPS data was calibrated to the C1s binding energy of 285.0 eV.

### Phase transition and photoresponse measurements of VO_2_ thin films

The VO_2_ thin film devices were fabricated using reactive ion etching, the metal contacts (15 nm Cr/200 nm Au as electrodes) were deposited by thermal evaporation. The thickness of as-grown VO_2_ thin film was ~ 1.2 μm with lateral dimensions 30 × 45 μm, respectively. Phase transition measurements were performed using a cryogenic probe station (Lake Shore TTPX) under a vacuum chamber with pressure about 1 × 10^−4^ Pa, the temperature was controlled by a Lake Shore 330 temperature controller with high temperature stability. Near infrared (NIR) response characteristics of the device were measured using a Keithley 2612 analyzer under dark and illuminated conditions. Light with a wavelength of 850 nm was switched on/off using a mechanical chopper (at a working frequency of 2 Hz). Optical attenuator was introduced to change the input power. The light was focused on the film with a 50 × objective (NA = 0.5), and the spot size of light is ∼1 μm.

## Data Availability

The data that support the findings of this study are available within the article.
